# Cell Density Impacts Population Activity in Human iPSC-Derived Neural Networks

**DOI:** 10.1523/ENEURO.0176-24.2026

**Published:** 2026-05-06

**Authors:** Yavuz Selim Uzun, Renata Santos, Maria C. Marchetto, Krishnan Padmanabhan

**Affiliations:** ^1^ Department of Physics and Astronomy, University of Rochester, Rochester, New York 14642; ^2^ Del Monte Institute for Neuroscience, University of Rochester School of Medicine, Rochester, New York 14642; ^3^Université Paris Cité, Institute of Psychiatry and Neuroscience of Paris (IPNP), INSERM U1266, Signaling Mechanisms in Neurological Disorders, Paris 75014, France; ^4^Institut des Sciences Biologiques, CNRS, Paris 75005, France; ^5^Department of Anthropology, University of California, San Diego, La Jolla, California 92093; ^6^ Department of Neuroscience, University of Rochester School of Medicine and Dentistry, Rochester, New York 14642; ^7^ Center for Visual Science, University of Rochester School of Medicine and Dentistry, Rochester, New York 14642; ^8^ Intellectual Development and Disability Research Center, University of Rochester School of Medicine and Dentistry, Rochester, New York 14642; ^9^ Department of Biomedical Engineering, Hajim School of Engineering, University of Rochester, Rochester, New York 14642

**Keywords:** iPSC neural cultures, MEA, network size, population coding

## Abstract

Multi-electrode recording of neuronal activity in cultures offer opportunities for understanding how the structure of a network gives rise to function. Neuronal cultures derived from human induced pluripotent stem cells (iPSCs) from male and female individuals are often plated at highly variable cell densities across studies, but its impact on neuronal activity remains poorly understood. We found that properties such as the mean firing rate of the individual cells, the pairwise correlations between cells, and the entropy of the population all changed significantly with changes in culture density. We used a maximum entropy model to capture the structure of the population activity using only the firing rates and correlations, and we found that the model performed best at the highest densities, suggesting that changes in activity reflected differences in structure of interactions between neurons across scales of complexity. Our work thus shows that culture density is an important experimental parameter that impacts neuronal activity in human iPSC-derived cultures. Additionally, our findings provide an analytical framework to study population activity in neuronal cultures including those from patient populations where a disease process may impact network activity.

## Significance Statement

iPSC-derived human neuronal cultures have been used to study the emergent patterns of population activity across development and in disease models. Across these studies, the culture density is one of the most variable experimental parameters, but it is unclear to what extent density affects population activity. By performing multi-electrode array (MEA) recordings of neuronal cultures at different densities, we identified how increasing the cellular density decreased the complexity of patterns of population activity.

## Introduction

Neuronal networks differentiated from induced pluripotent stem cells (iPSCs) have emerged as one of the most promising systems for modeling a vast array of human diseases ranging from neurodevelopmental disorders to neurodegeneration (Ardhanareeswaran et al., 2017; Rivetti di Val Cervo et al., 2021; Whiteley et al., 2022; Wang et al., 2023). Neuronal cultures are especially compelling because they allow for study of disease pathology across multiple scales of complexity, such as gene expression, cellular physiology, and synaptic transmission. Each level of analysis provides a different lens within which to view disease processes and therefore identify mechanisms and potential areas of intervention (Chiaradia and Lancaster, 2020; Kim et al., 2020; Andrews and Kriegstein, 2022; Eichmüller and Knoblich, 2022).

One common way to functionally assay activity in neuronal cultures is using multi-electrode arrays (MEAs), where grids of electrodes measure the extracellular spiking activity from populations. MEA recordings of neuronal networks have been used as functional readouts of different networks including from neurotypical individuals and from patient populations as well as a platform for drug discovery and drug testing (Pelkonen et al., 2021; Vadodaria et al., 2021; Pré et al., 2026). Although MEAs are ubiquitous in functional measures of human iPSC-derived neuronal populations, many culture parameters, such as density, vary log orders across studies (Marchetto et al., 2017; Amatya et al., 2019; Fair et al., 2020; Sandoval et al., 2024).

Cell density is one such parameter that is known to be important. For example, in mouse cultures, networks containing <500 neurons/mm^2^ had ∼400 synapses per neuron while higher-density cultures, such as ones with 4,500 neurons/mm^2^ had far fewer synapses per neuron, 150 per cell (Cullen et al., 2010). While cell density varies across an array of iPSC-derived neuronal culture experiments (Marchetto et al., 2019; Mok et al., 2022; McCready et al., 2025; Pré et al., 2026), it is unknown whether density may affect neuronal activity in these human model systems. Understanding the relationship between density and neuronal activity could also provide insight into fundamental questions of network function and neural activity, particularly in the context of disease modeling using human iPSC-derived cultures from patient populations.

To address these questions, we measured activity of human iPSC-derived neuronal cultures across different neuronal densities using MEAs. First, we found that while some measures of neuronal activity, such as firing rate, changed in a systematic way with changes in cell density, others such as the pairwise correlations between neurons did not change. Interestingly, when we examined the activity at the population level, we found that the entropy of the population spiking activity decreased with increased culture density. Furthermore, a statistical model which predicted the activity of the population from the firing rate and the pairwise correlations performed better as cell density increased, showing that population activity was less diverse as the density of the neuronal cultures increased, implying that the complexity of the networks decreases as the culture density is increased. Taken together, these data suggest that properties of neuronal network organization change in fundamental different ways simply by changing the density of the neurons in the network. Our results provide one analytical framework with which population neuronal activity can be studied and compared across different experimental groups. Additionally, the analyses presented here can be invaluable for researchers studying in vitro human neuronal activity patterns for the purposes of disease modeling.

## Materials and Methods

### iPSC lines and neuronal differentiation

The two iPSC lines used in this study were derived from healthy controls, one female and one male, as previously described (Marchetto et al., 2010): WT-33 (RRID:CVCL_HA45) and WT-126 (RRID:CVCL_YL15). Neurons were differentiated from iPSCs through intermediate generation of neural progenitor cells (NPCs) as described (Marchetto et al., 2019). Briefly, iPSC colonies were dissociated from Matrigel (Corning)-coated plates with 1 mg/ml collagenase IV (Thermo Fisher Scientific) and plated onto low-adherence plates in mTeSR1 medium (STEMCELL) to form a suspension of embryoid bodies (EBs). The day after, the medium was changed to NPC medium: DMEM/F12 medium with GlutaMAX with 1× N2 supplement and 1× B27 supplement (all from Thermo Fisher Scientific) plus 500 ng/ml recombinant human noggin (Proteintech). After 10 d in suspension, the EBs were plated onto 10 µg/ml poly-ʟ-ornithine (PLO, Sigma-Aldrich) and 5 µg/ml laminin (Invitrogen) coated plates and maintained in NPC medium plus 500 ng/ml noggin and 1 µg/ml laminin. For plate coating, PLO solution in PBS was incubated overnight at room temperature, washed thoroughly three times with PBS, and followed by incubation for 2 h at 37°C with laminin solution in PBS. The neural rosettes were collected after 7 d, gently dissociated with Accutase (STEMCELL) and plated in PLO-laminin-coated dishes. This NPC population was expanded using the NPC medium plus 10 ng/ml FGF2 (Joint Protein Central) and 1 µg/ml laminin. To differentiate the NPCs into neurons, the cells were plated into PLO-laminin-coated dishes and cultured in DMEM/F12 GlutaMAX supplemented with 1× N2 and 1× B27, 1 µg/ml laminin, 20 ng/ml BDNF (PeproTech), 20 ng/ml GDNF (PeproTech), 0.2 µM ascorbic acid (STEMCELL), 1 µM retinoic acid (STEMCELL), and 500 mg/ml cyclic AMP (Tocris), with half-medium changes three times a week. This protocol produces terminally differentiated neurons and glia as has been previously described (Marchetto et al., 2013; Maroof et al., 2013; Sandoval et al., 2024).

### Immunocytochemistry

Cells were fixed in 4% paraformaldehyde (Sigma-Aldrich) for 10 min at room temperature. Antigen blocking and cell permeabilization were done using 5% horse serum and 0.3% Triton X-100 in PBS for 20 min at room temperature. Primary antibodies prepared in 5% horse serum were incubated overnight at 4°C, washed with PBS, and incubated with secondary antibodies (1:500, Jackson Laboratory) for 1 h at room temperature. The primary antibodies used were the chicken anti-MAP2 polyclonal (1:1,000, Abcam 5392), rabbit anti-GFAP polyclonal (1:5,000, Thermo Fisher Scientific 01670276). Cells were counterstained with DAPI for cell nuclei visualization.

### MEA recordings

Twelve-well MEA plates from Axion Biosystems were used to record electrical activity of neurons. Each well contains 64 electrodes arranged in an 8 × 8 grid spaced by 300 µm, with an electrode contact diameter of 50 µm and a recording area of 2.1 mm × 2.1 mm. The NPCs were plated in tetraplicate in 10 µg/ml PLO 5 µg/ml laminin-coated plates at different densities and neuronal differentiation started the day after. Half-media was changed three times a week and measurements were taken before change. Extracellular recordings were performed in a Maestro MEA system and AxIS software (Axion Biosystems) with voltages recorded at a frequency of 12.5 kHz. Following 5 min of plate rest time in the instrument chamber with controlled temperature and CO_2_ levels, recordings were performed for 15 min.

### Spike sorting

Spike sorting was performed in accordance with recent recommendations aimed at improving rigor and reproducibility in iPSC-derived neuronal research (Sandoval et al., 2024). The Maestro MEA system's hardware bandpass was 0.01 Hz–5 kHz, and no additional filtering was done at the recording stage. To isolate spiking, we applied a bandpass filter (500–3,500 Hz) using the “butter” function of MATLAB followed by a 60-cycle notch filter. Four-second padding was applied to the recording during the filtering process to prevent edge effects. We identified event crossings that were greater than 5 standard deviations from the mean as putative spikes. While the choice of threshold crossing over a range from 4.5 to 5.5 standard deviations altered the number of units and spikes detected, thus affecting the specific values of firing rates and correlation across this range, the conclusions of our study did not change and were thus robust to parameter choice. From these events, we eliminated high frequency periodic oscillations that corresponded to noise based on their symmetry using a correlation threshold and an absolute value difference peak and trough of 8. Once data had been preprocessed to spike sort, we first used event times in each channel to extract waveform information in the neighboring channels. Waveforms associated with each event were concatenated, and principal component analysis was performed to project spiking activity into a low dimensional space. To cluster spiking activity, we performed expectation maximization (EM) on a Gaussian mixture model of the low dimensional projection as has been described previously (Lewicki, 1998; Rossant et al., 2016). Once putative single units had been identified, we examined the mean waveforms of sorted clusters and grouped together those whose waveform shape correlation was greater than 0.8 to prevent overclustering. We confirmed that our results were robust to different correlations that may affect over/under sorting of spikes as described (Lewicki, 1998). Once units were isolated, we further validated them by ensuring that isolated units had less than 5% of their spikes occur in interspike interval lower than 2.5 ms. This interspike interval selection criterion ensured that subsequent analyses were not affected by the absolute refractory period of neurons. Additionally, putative units where the isolated waveform contained multiple peaks that correspond to compound spikes were excluded from this analysis. While previous studies have examined bursting in iPSC-derived recordings, this measure of activity happens on time scales of several hundred milliseconds and as such, the temporal window that experiments use to define a burst varies from study to study (Maeda et al., 1995; Wagenaar et al., 2006a,b; Marchetto et al., 2017). Furthermore, these studies vary significantly in what aspect of activity constitutes a burst, such as the number of units that fire within a time window or the number of channels on which spiking activity occurs. As a complementary approach, our studies focused on collective neuronal activity on a much shorter time scale, that of millisecond temporal resolution.

To further ensure the robustness of our work, we also used a different spike sorting algorithm and superparamagnetic clustering (Quiroga et al., 2005; Minxha et al., 2020) and found that the central findings of our manuscript were not affected. The different algorithms we used differentially sort spikes both within a given channel and across channels over a range of thresholds, with different selection criterion, and with different features of the statistics of spiking; central results of this manuscript were significant across all these different parameters and sorting methods. All analyses were performed in MATLAB.

### Estimating the position of units using correlated activity at the neighboring channels for visualization

Spiking from a given unit may be detected across multiple channels, particularly when the dendritic arbors span a large region. To visualize waveforms, we identified the waveform shape of each unit across the different channels. We then created a weighted sum of the waveform shape (the integral of the absolute value of the waveform shapes on each channel where the unit was detected). This allowed us to display a putative location of the soma. As the integral of the waveform shape weights the shape of the waveform rather than use a scaling factor, this facilitated an estimate of putative locations of the soma which was used for visualization and for illustration of spike sorting.

### Stationarity of recordings

Only recordings where spiking activity was stationary throughout the recording duration were used in all analyses. Stationarity was determined in three ways. First, by dividing spikes from the recording into two-halves and calculating properties of spiking such as the spike rate and the waveform. Only recordings where the firing rates of the unit did not change between the first and second half of the recording were included. Second, we calculated a continuous firing using a sliding window (100 ms window size, 5 ms step size) over the entire recording and then calculated the distributions of firing rates across two-halves of the recordings. These distributions were then compared with each other using the Kullback–Leibler divergence (KL-D) and then compared the distributions of firing rates in one-half of a given channel to another channel using the Welch ANOVA test applied for calculating the significance of the difference between KL-D distributions.

To quantify the spatial distribution of putative units across the culture, we first divided the total recording space into square regions and calculated the probabilities of units located within each square region. We then calculated the entropy of this probability distribution across different square sizes over a 2 log order scale (in units of µm). This entropy was normalized by the maximum entropy.

### Pairwise correlation

To calculate the pairwise similarity between the firing patterns of two units, the binarized time series of spike trains were convolved with a 25 ms Gaussian and the Pearson’s correlation coefficient was calculated for all unit pairs.

### Count and word entropies

To quantify entropy, we discretized time series into bins with a 10 ms time resolution such that if a spike occurred in that window, it was registered as a 1, and if not, it was represented as a 0. More than 95% of bins had only 1 spike consistent with bin sizes used to calculate entropy in other studies (Osborne et al., 2008). In the remaining 5% of bins that contained more than one spike, we represented this as 1 (irrespective of the number of events). Count entropy was calculated as the number of units that simultaneously spike in at any given moment of time, regardless of the identity of units that spike. Word entropy calculates the distribution of binary patterns of 1 and 0 s across the population, preserving information about unit identity. As we recorded different numbers of units simultaneously across different experiments, we computed entropy by subsampling the across different population sizes (*K* = 2–18 units). For any given population, we ensured the stability of the entropy calculation by bootstrapping with replacement.

### Maximum entropy

We used Maoz's maxent_toolbox for the maximum entropy calculations (Maoz and Schneidman, 2017). This package generates the maximum entropy distribution for a given training data utilizing the set of constraints applied. The generated distribution has the minimal amount of structure other than the constraints we applied. As with the entropy calculation, we subsampled the units across different population sizes (*K* = 2–14 units). For the second-order maximum entropy model, we used firing rate and pairwise interactions parameters as constraints in the analysis. To do this, data was first divided into two parts; half the data was used to determine the statistical parameters underlying features of population activity, specifically the firing rate of individual cells and pairwise interactions between all pairs of neurons. Predicted distributions of patterns of activity were generated from the parameters and compared with the remaining half empirical data. Results were evaluated by comparing the empirical and the synthetic data using the KL-D.

### Statistics and rigor

To evaluate the significance for the findings, for all data, distributions were examined to test for normality with the Kolmogorov–Smirnov test, and variances were compared with the Levene absolute test. As all data were not normally distributed and arose from different variances, the statistical significance was determined with the two-sided Brunner–Munzel test with Bonferroni’s correction for multiple comparisons.

### Code accessibility

The analysis was performed with MATLAB 2022b on Windows operating system. The code described in the paper is freely available online at https://github.com/krishnanURMC/Uzun_eNeuro_2026. The code is available as Extended Data.

10.1523/ENEURO.0176-24.2026.d1 Data 1 All analysis code for this manuscript can be found at https://github.com/krishnanURMC/Uzun_eNeuro_2026 Download Data 1, ZIP file.

## Results

### Generation of iPSC-derived neural networks of different size

**Figure 1.**
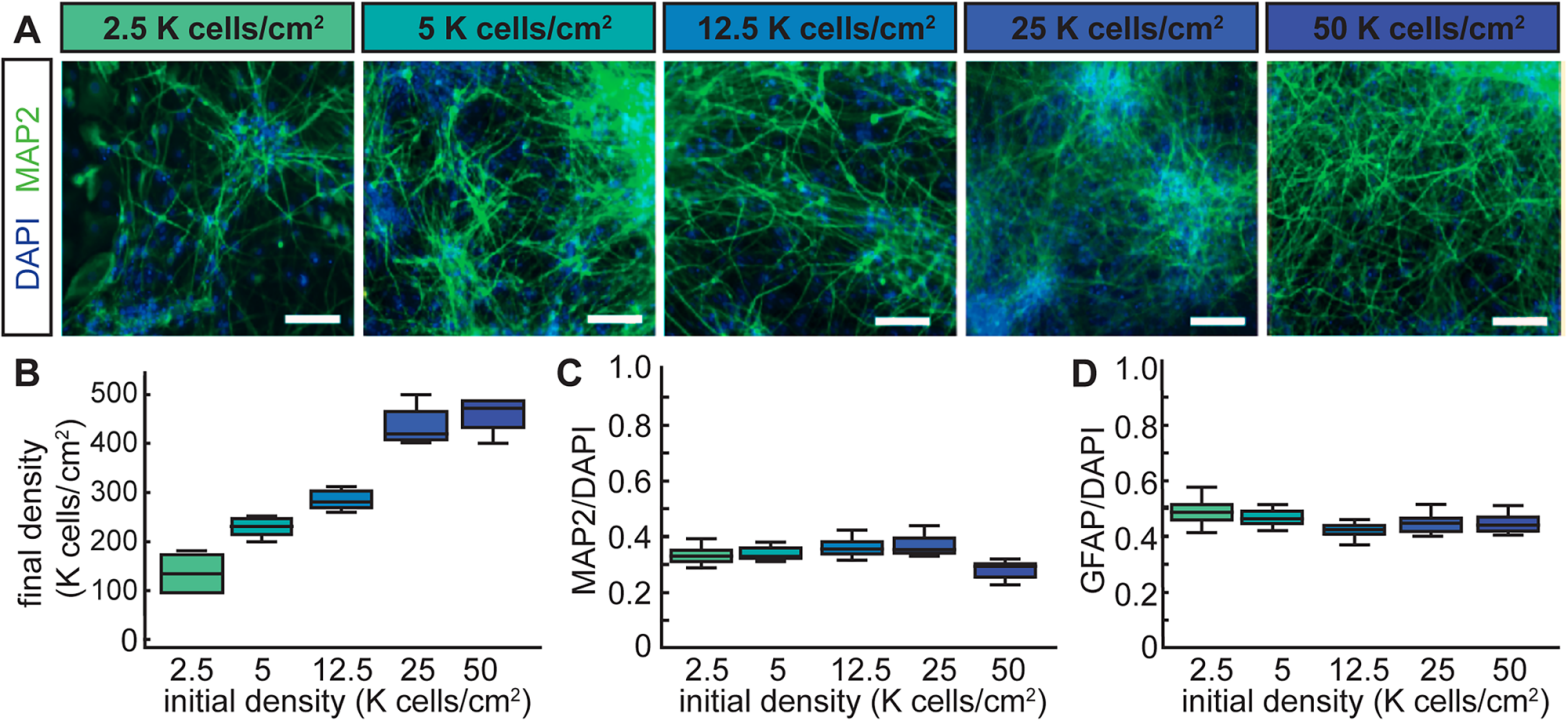
Generation of iPSC-derived neuronal networks of different sizes. ***A***, Representative images of immunohistochemistry for the neuronal markers MAP2 (green) at 8 weeks. Nuclei of all cells were counterstained with DAPI (blue). Scale bar, 100 µm. ***B***, Final number of cells in K cells/cm^2^ at 8 weeks as a function of the initial number of plated cells (2.5, 5, 12.5, 25, and 50 K cells/cm^2^). Boxplot shows data from 4 wells. ***C–E***, Quantifications at 8 weeks of cells expressing the neuronal markers MAP2 (***C***) and the astrocytic markers GFAP (***D***) as a function of initial cell density. Boxplots show data from 7–12 randomized images.

To study the relationship between neuronal density and network organization, we differentiated human iPSC-derived NPCs into neuronal and glia over the course of 8 weeks and recorded spontaneous extracellular activity using MEA for 250 h (*N* = 792 recordings). Cultures were plated at five different densities (2.5, 5, 12.5, 25, and 50 K cells/cm^2^; Fig. 1*A*). Cells were counted on the last day of recording and showed a significant correlation (*R* = 0.87, *p* < 0.005) between the plating density and the final cell counts (Fig. 1*B*). This type of protocol for neuronal differentiation is widely used in the human iPSC field across multiple labs and the plating densities selected corresponded to densities used across prior studies allowing us to determine how plating density affects patterns of neuronal activity.

To determine how culture density affected the molecular identity of cells, we used immunohistochemistry to determine the composition of the populations across all cultures. We found that there was no significant difference in the percentage of MAP2-positive cells corresponding to the number of neurons in the culture, nor did we observe any significant difference between the number of GFAP-positive cells corresponding to the astrocytes across all densities (Fig. 1*C*,*D*). These data indicate that the overall cellular composition of neurons and glia in the cultures was similar across the different densities.

### Spike detection and neuron identification

**Figure 2.**
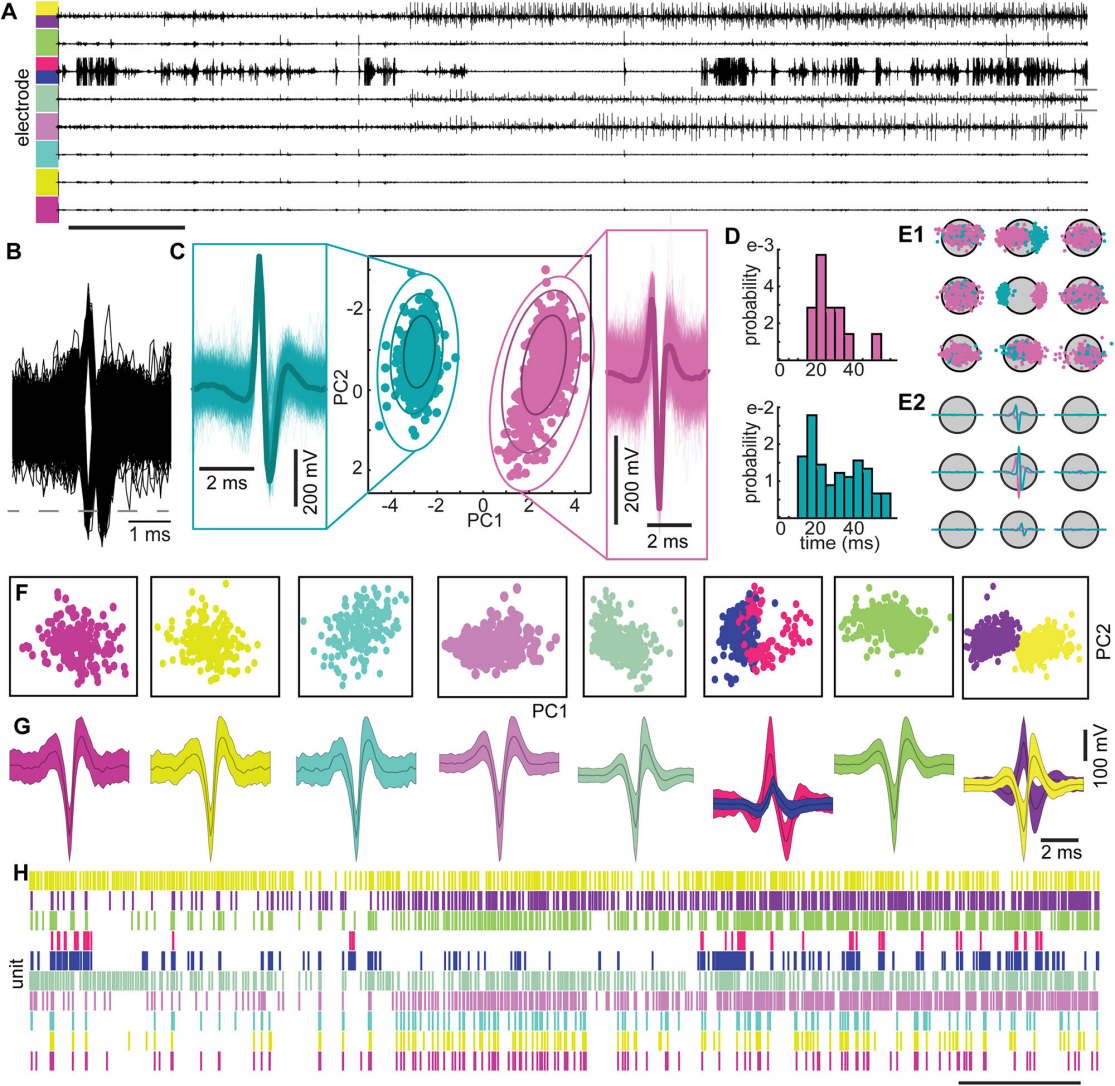
***A***, Example of raw recording of 8 channels from the total 64 channels recorded in a single low-density culture showing clear spiking activity across the channels. Colors correspond to units identified following spike sorting in each channel. ***B***, Waveforms detected at a single channel. ***C***, Representation of the waveforms in two-dimensional PCA space. Color-coding shows the sorted units found by the Gaussian mixture model clustering method. Waveforms shown at the sides depicts the sorted units with this method. ***D***, Corresponding interspike interval distribution of the sorted units. ***E1***, Gray dots represent the recording channels, and center channel is where the units were assigned to and the other channels are the surrounding channels. We show the color-coded PCA representation of the detected units in the surrounding channels. ***E2***, The mean waveforms of the sorted units detected at the surrounding channels. ***F***, ***G***, Lower dimensional representations and average waveforms of the units detected at the voltage traces we showed in panel ***A***. ***H***, Raster plot of the activity of the detected units from ***A*** following spike sorting. This allowed segregation of the activity from multiple units recorded on a single channel and to isolate the activity of a single unit that was recorded across multiple channels.

To characterize the neurophysiological properties of neuronal cultures, we measured extracellular spiking using low-impedance 64-channel (8 × 8 grid) MEAs. Across multiple channels, we observed complex patterns of neuronal activity (Fig. 2*A*), wherein activity from multiple individual neurons (or units) could be observed on each channel (Fig. 2*B*). To separate the activity on each channel into spiking from individual units, we implemented a well-established spike sorting algorithm (see Materials and Methods) that has been previous used from both in vivo and in vitro recordings (Lewicki, 1998; Hales et al., 2012; Rossant et al., 2016; Marchetto et al., 2017; Chockanathan and Padmanabhan, 2021). Spike waveforms were projected into a low dimensional space defined by the principal components of the covariance matrix (Fig. 2*C*) and an expectation maximization of a mixtures of Gaussians (EM-MG) model was used to isolate waveforms corresponding to individual units (Fig. 2*C*, colors correspond to units). We further confirmed that the waveforms we identified were from single units by ensuring that all units had fewer than 5% of spikes with less than a 5 ms interspike interval (Fig. 2*D*). Additionally, we separated single unit activity into individual neurons across multiple neighboring channels (Fig. 2*E*). As a result, we were able to isolate multiple neurons wherein the waveforms formed distinct clusters (Fig. 2*F*) corresponding to unique spike waveform shapes (Fig. 2*G*) that allowed us to track the population activity of ensembles across the neural cultures (Fig. 2*H*).

**Figure 3.**
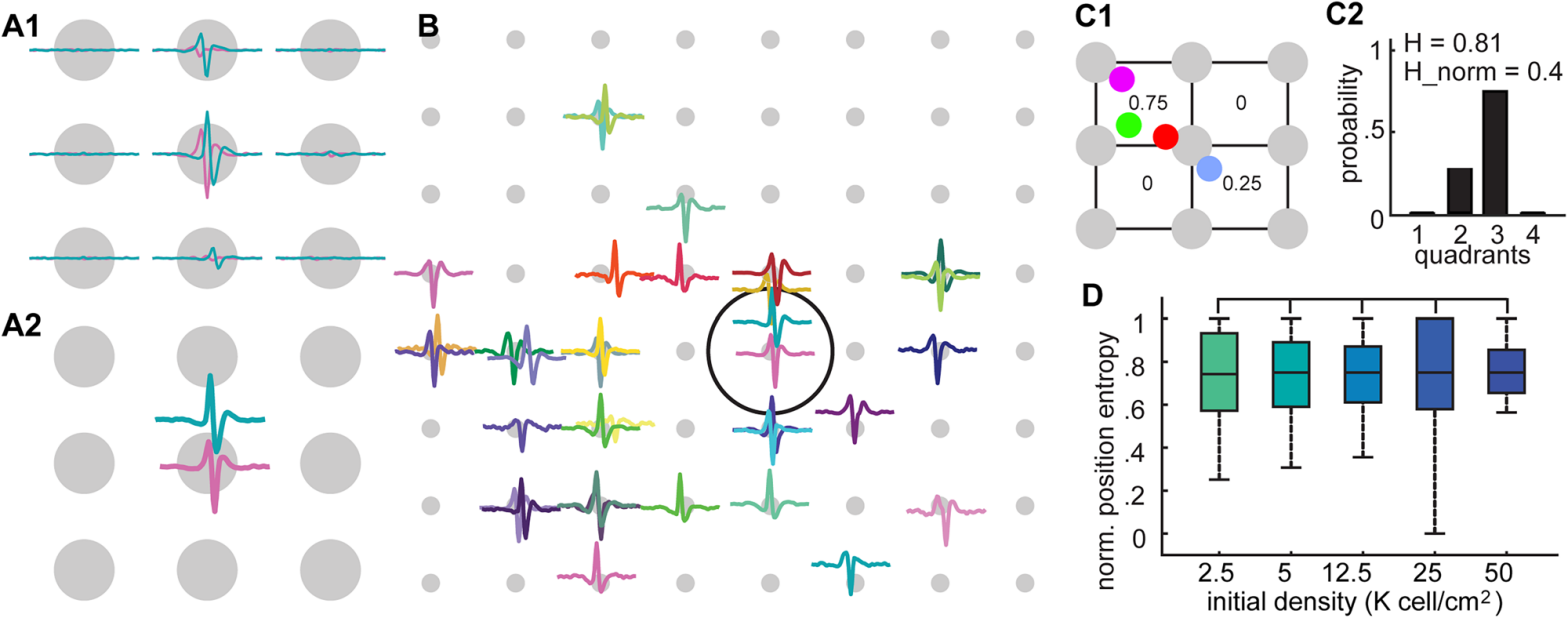
Location of the units were found by triangulation method and used to compare the spatial distribution of the units coming from different density categories. ***A1*–*2***, We found a more precise location of units utilizing the voltage amplitudes in the surrounding channels. Gray dots representing the recording channels where the center channel is the channel which units were assigned to. Waveform of each unit is represented with a different color. In ***A1***, we showed an example of unit waveforms in the surrounding channels. ***A2***, Each waveform shows the predicted unit location, found by weighing the channel positions with the waveform amplitude in the corresponding channel. ***B***, Shows an example triangulation of the unit positions in a recording. Panels ***C*** and ***D*** shows how positional entropy characterizes the homogeneity of the spatial distribution of the neurons. ***C1***, Gray dots show channel positions and colored dots show unit positions on the physical map (which are different from the units in ***B***). Squares drawn with black lines show the edges of each quadrant. ***C2***, We showed corresponding probability distribution for finding a unit inside in each quadrant. H is the positional entropy, and H_norm is the normalized positional entropy which is calculated by dividing H with the positional entropy of spatially the most random case (Materials and Methods). The size of the quadrants drawn in panel ***C1*** is arbitrary and can characterize the homogeneity of the physical distribution at different length scales when it gets bigger or smaller. ***D***, Normalized positional entropy distributions were not significantly different for different density categories, across multiple length scales. Here we showed the resulting distributions for each density measured on the scale of 1 mm (densities 2.5, 5, 12.5, 25, and 50 K cells/cm^2^ with 96, 126, 74, 102, and 52 neural population over all weeks in vitro, *p* > 0.0033. Statistics: one-sided Brunner–Munzel test with Bonferroni’s correction.).

**Figure 4.**
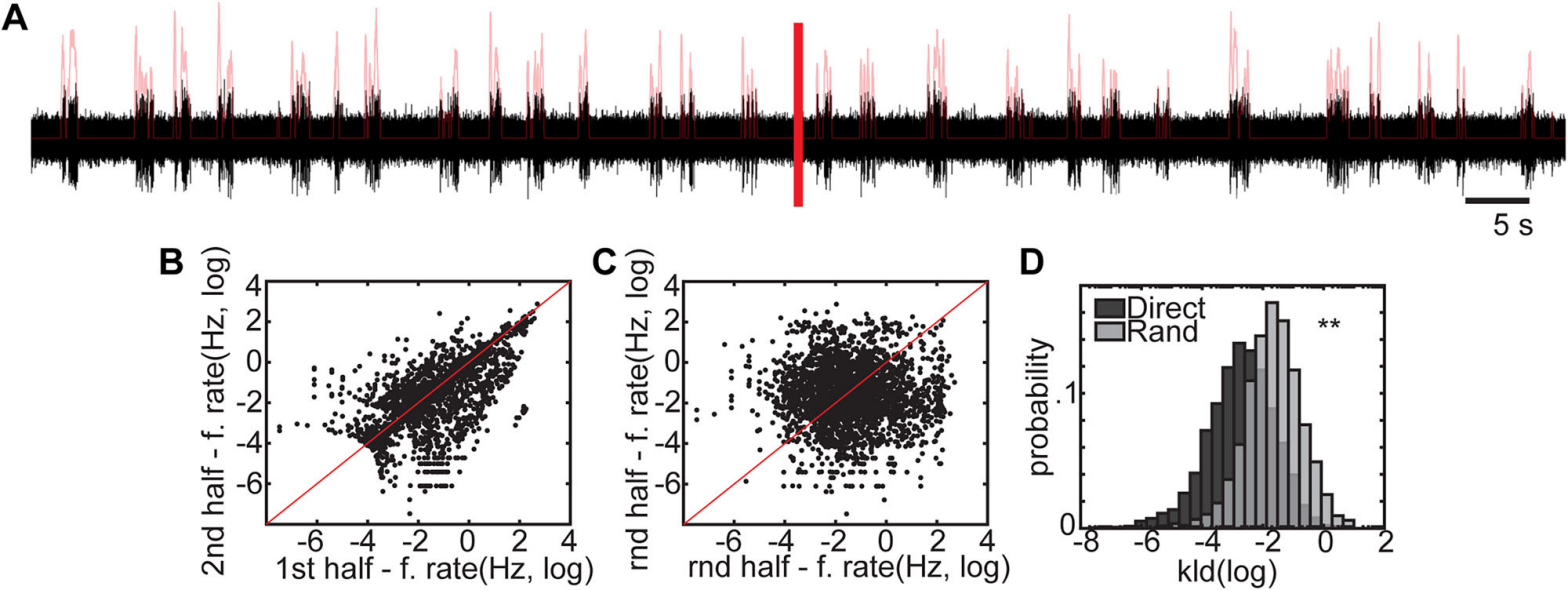
Stationarity of the firing rate. ***A***, Example voltage trace in black. Red line in the center is showing the middle of the recording. We showed the binarized and convolved time series with light red trace (moving mean firing rate) overlapping the black voltage trace. ***B***, Comparison of the firing rates of first and second halves of the recordings. ***C***, Comparison of firing rates of the halves of random units. Red line panels ***B*** and ***C*** is showing the unity line. ***D***, Moving average firing rate distribution calculated with a 100 ms moving window over the convolved time series (light red trace in panel ***A***). We quantified how different were the distributions coming from the first half of the recording and the second half of the recording using KL-D. Panel ***D*** shows the probability of getting KL-D values when comparing the distributions of first and second half of the same unit (dark gray) and the distributions of random halves (light gray). The KL-D values were significantly lower when compared the halves of the same unit instead of halves of random units. (*N* = 2,880 pairs, *p* < 0.005. Statistics: Welch test).

As the activity from individual units was observed across multiple channels (Fig. 3*A1*), we used this information to triangulate a location for each unit (Fig. 3*A2*) within MEA array field (Fig. 3*B*). This allowed us to determine if the spatial distribution of recorded units was the same across different densities. To do this, we measured the fraction of units within small quadrants (Fig. 3*C1*) and quantified the distribution of these fractions (Fig. 3*C2*) as a measure of entropy. We found no significant difference in the spatial distribution of recorded units across all densities (Fig. 3*D*) at different scales from 100 µm to 1 mm further confirming that the differences in neuronal activity across populations were not due to factors such as different sampling of neurons in space. Additionally, as our recordings spanned durations of up to 1 h, we sought to confirm the absence of systematic drift or changes in the firing patterns of the recorded neurons, thereby ensuring that the statistics of activity remained stationary throughout the recording period. To do this, we compared firing rates for each unit in the first and second halves of the recording period (Materials and Methods; Fig. 4*A*). As shown in Figure 4*B*, firing rates of the same unit across halves closely aligned along the unity line (red), indicating consistent activity across time. By comparison, when we examined the firing rates of two-halves of the recordings from random units (Fig. 4*C*), we found no significant correlation, suggesting that the firing rate statistics of our recordings were stable over the duration of the experiments.

To further quantify stationarity, we generated histograms of moving average firing rates for the first and second half of the recordings and then calculated the difference in the distributions as measured by the KL-D (Fig. 4*D*) Smaller KL-D values correspond to distributions that are more similar and we found that KL-D values were significantly lower in same-unit comparisons (Fig. 4*D*, dark gray) as compared with randomized pairs (Fig. 4*D*, light gray), confirming that unit firing was stable over time (*p* < 0.005).

### Microscopic features of network activity change with network density

**Figure 5.**
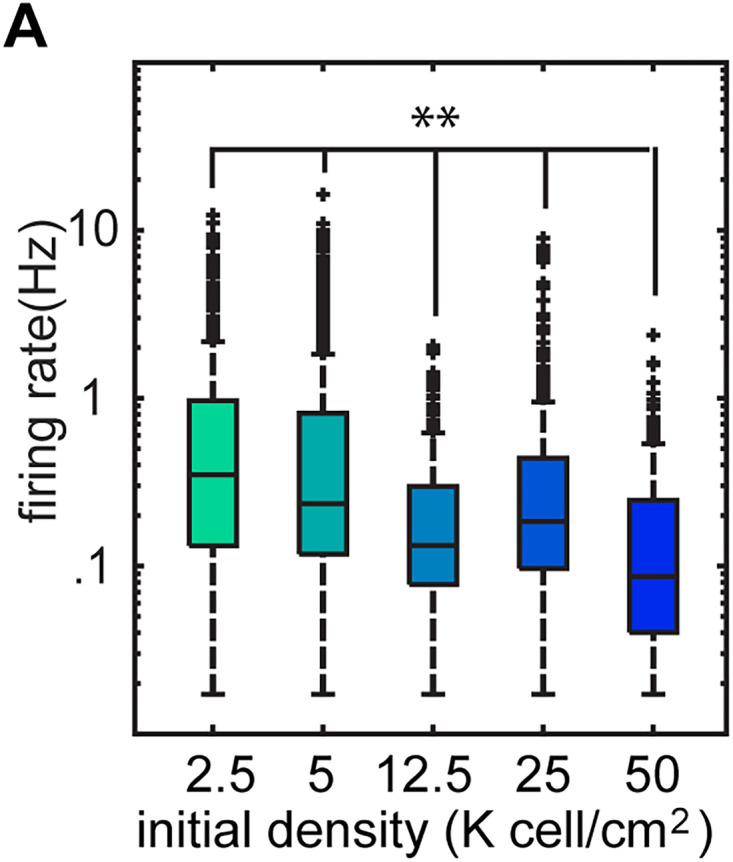
Firing rates of networks of different densities vary significantly. The distribution of firing rates of cultures with initial densities 2.5, 5, 12.5, 25, 50 K cells/cm^2^. A significant difference between all category pairs (initial densities 2.5, 5, 12.5, 25, 50 K cells/cm^2^ with 552, 1,039, 468, 554, 267 units over all weeks in vitro, *p* < 0.0033. Statistics: two-sided Brunner–Munzel test with Bonferroni’s correction). Firing rate decreased with increasing cell density.

**Figure 6.**
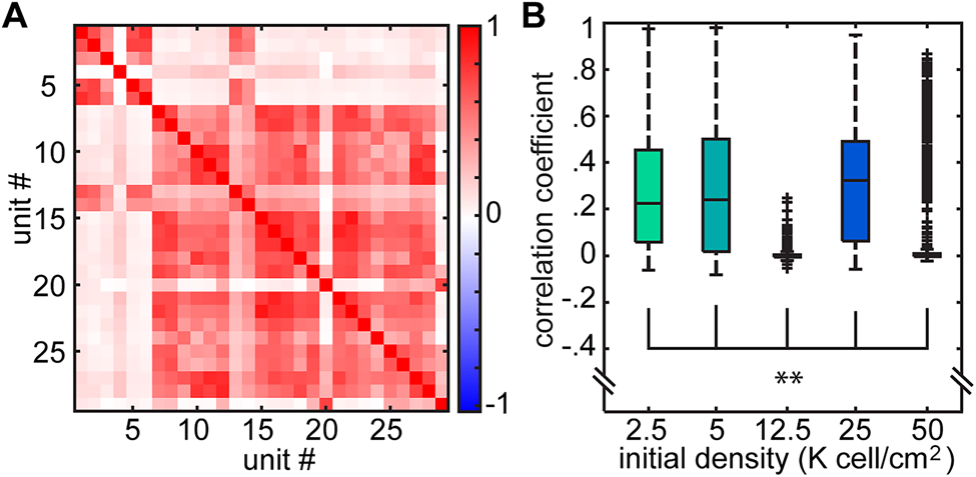
Pairwise correlation cannot reveal heterogeneities in functional connectivity across different density categories. ***A***, Example pairwise correlation structure of the activities. ***B***, Box plot illustrates the distribution of pairwise correlation coefficients for each density category. While no trend is apparent across the categories, the distributions were significantly different from each other (initial density categories were 2.5, 5, 12.5, 25, 50 K cells/cm^2^ with 4,161, 9,597, 1,984, 4,021, 1,118 unit pairs over all weeks in vitro), *p* < 0.0033. Statistics: two-sided Brunner–Munzel test with Bonferroni’s correction.

The simplest measure of unit activity that one can measure is the firing rate of individual neurons across different densities. When we examined the firing rate, either as the mean firing rate or the median firing rate, we found that spike rate decreased with increased density, suggesting that excitability and spontaneous firing scaled with the size of the networks in the culture (Fig. 5). In addition to firing rate, the pairwise correlations between the individual units have been used to assess both circuit organization and network computation (Singer, 1999; Salinas and Sejnowski, 2001; Perin et al., 2011; Tremblay et al., 2016). Pairwise correlations measure the degree of similarity between the firing patterns of pairs of cells and can reflect features of circuit organization ranging from the degree to which the cells are connected to the extent to which a pair of cells receives correlated input from some presynaptic population (Cohen and Kohn, 2011; Litwin-Kumar et al., 2011). Figure 6*A* shows the pairwise correlations across a population of simultaneous recorded units. While a large group of cells fire synchronously across the population, there are numerous examples of cells that have patterns of activity that are distinct from the overall population; differences reflected in the different pairwise correlations (Fig. 6*A*). We found a significant difference in the distributions of correlations for the different densities (Fig. 6*B*), suggesting that the network organization (as assessed by the covariance of firing) was different as a function of the overall density of the network. Taken together, we found that both firing rate and correlation changed as a function of the density of the cultures, suggesting that these microscopic measures of the individual neuron's firing and degree of synchrony may be influencing the overall global structure of the network.

### Macroscopic organization of population activity differs with network density

To understand how these myriad changes in both the activity of cells and their functional interactions with one another govern the overall structure of population activity, we turned to a measure of macroscopic or global population activity called entropy (de Ruyter van Steveninck et al., 1997; Abbott et al., 2005; Averbeck et al., 2006; Vyas et al., 2020). For example, a network with highly synchronous neurons that all fire together will be in one of two states, either all the cells will be quiescent or all the cells will spike. This would be an example of a low entropy network, where the diversity of activity patterns across the population is small. In contrast, where every combination of activity patterns across the population is equally likely, the entropy will be high. In practice, networks rarely exist in these two extremes. Instead, these examples provide an intuition as to how entropy may identify nuanced differences in network organization that may not be discernible using correlation alone.

**Figure 7.**
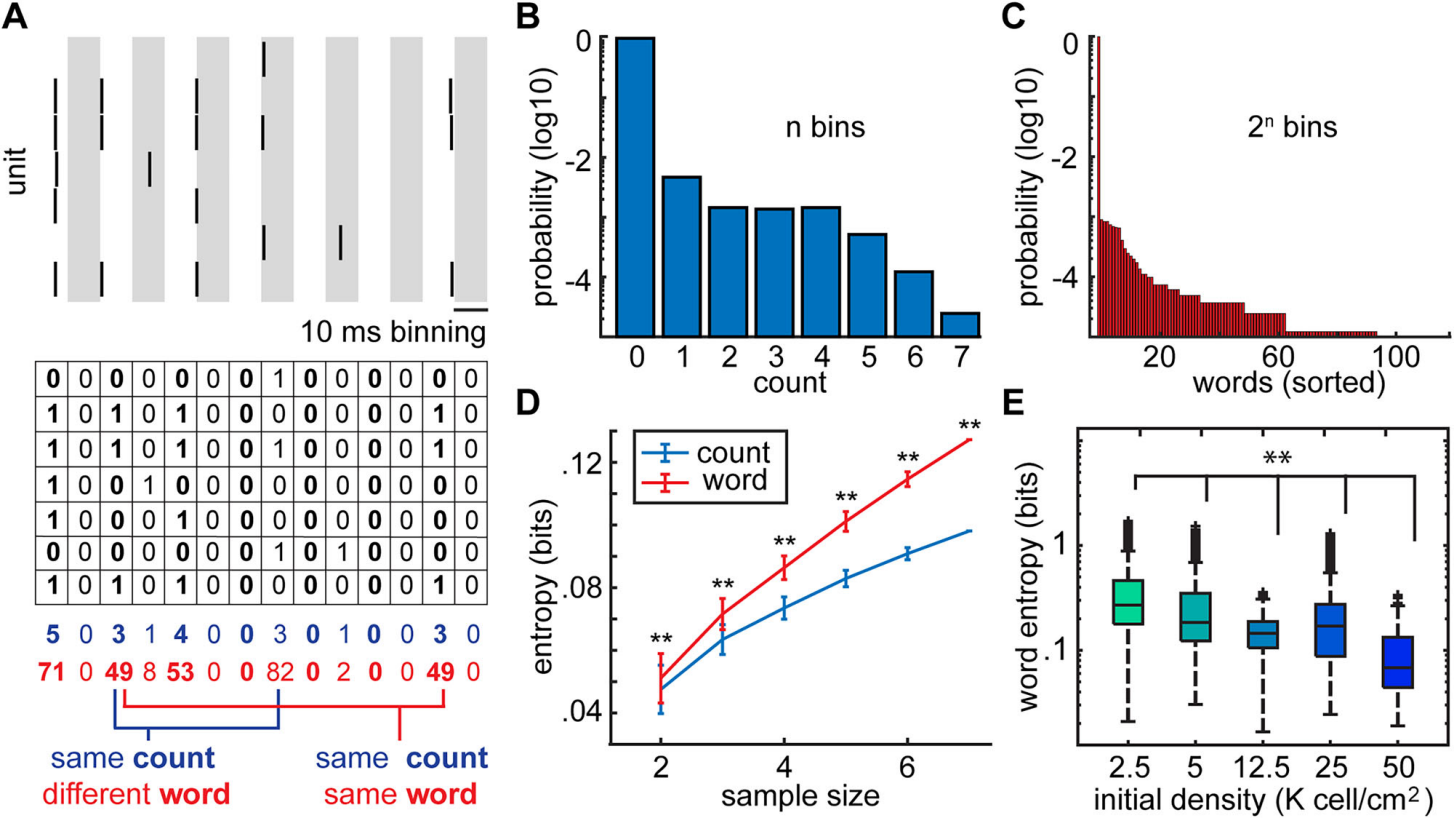
Characterization of population activity using entropy reveals the relationship between the initial density and the activity complexity. ***A***, On top is an example of spiking across a population of seven neurons. The activity is binned into 10 ms intervals (interleaved with gray shading to show bins). On the bottom, the spikes were binarized into patterns of 1 s (1 or more action potentials within a bin) and 0 s (no action potentials in each bin). For each 10 ms window was calculated the count (blue) corresponding to the number of units active and the word (red) corresponding to the pattern of neuronal activation. For 7 units, there can be 8 different patterns of activity when measuring counts and 128 different patterns of activity when measuring words. ***B***, Distribution of counts for the population in ***A*** across all time represents the probability of seeing any given pattern of activity. The distribution of counts corresponds most similarly to measures of synchrony and bursting. ***C***, Distribution of words for the population in ***A*** across all recording time shows the likelihood of seeing any combination/pattern of neuronal activity. ***D***, Word entropy (red) is higher than count entropy (blue) across all population size (different numbers of neurons) showing that word entropy captures more of the richness of population activity than count entropy [100 subsamples of this neuronal population (*N* = 2–7 neurons) where a total of 7 neurons were recorded]. (*p* < 0.005 for all samples with multiple pairwise-comparison correction; *p* = 2.6 × 10^−4^, 8.3 × 10^−22^, 3.4 × 10^−33^, 2.4 × 10^−34^, 1.9 × 10^−34^, 9.6 × 10^−41^ for *k* = 2, 3, 4, 5, 6, 7 respectively; significance was measured with Wilcoxon rank sum test). ***E***, Word entropy distributions of all initial density category pairs were significantly different (initial densities 2.5, 5, 12.5, 25, 50 K cells/cm^2^ with 1,039, 2,165, 1,197, 769, 638 subsampled populations over all weeks in vitro, *p* < 0.0033). Statistics: two-sided Brunner–Munzel test with Bonferroni’s correction. Word entropy decreased with increasing cell density.

To quantify the entropy of the cultures with varying densities, time series were binarized in 10 ms bins of time, such that if a spike occurred within this window, it was registered a value of 1 and if no spike occurred, it was registered as a 0 (Fig. 7*A*). This bin size ensured that over 95% of the 10 ms windows where action potentials were detected had only one event in the bin and consistent with bin sizes used to calculate entropy in other studies of population activity (Osborne et al., 2008). To illustrate how entropy might pick up the differences in population activity that pairwise correlations may not, we quantified a metric called the count entropy (which measures how many neurons fire at the same time and can be thought of as a proxy for synchrony; Maeda et al., 1995) and a metric called the word entropy (which described the distribution of all the combinations of firing patterns within a given time window). In this example, whereas the count entropy values would be the same across different periods of time, corresponding to similar degrees of correlated bursting, the measure of the word entropy at those same periods reveals the differences in the population activity structure. When we examined the probability distribution of counts (Fig. 7*B*) and of words (Fig. 7*C*), we found that quantifying networks in terms of word patterns was more informative. Indeed, when we calculated the count entropy versus the word entropy for this example (Fig. 7*D*) and across all experiments, we found that word entropy was always significantly higher than count entropy in bits, and this held across networks with different numbers of neurons (sampling size; Fig. 7*D*). When we quantified the word entropy (which we shall refer to simply as entropy) across the different densities, we found that entropy decreased significantly as culture density increased (Fig. 7*E*). Rather unexpectedly, we found that the diversity of patterns that networks generated decreased as the density of the cells in the network increased.

**Figure 8.**
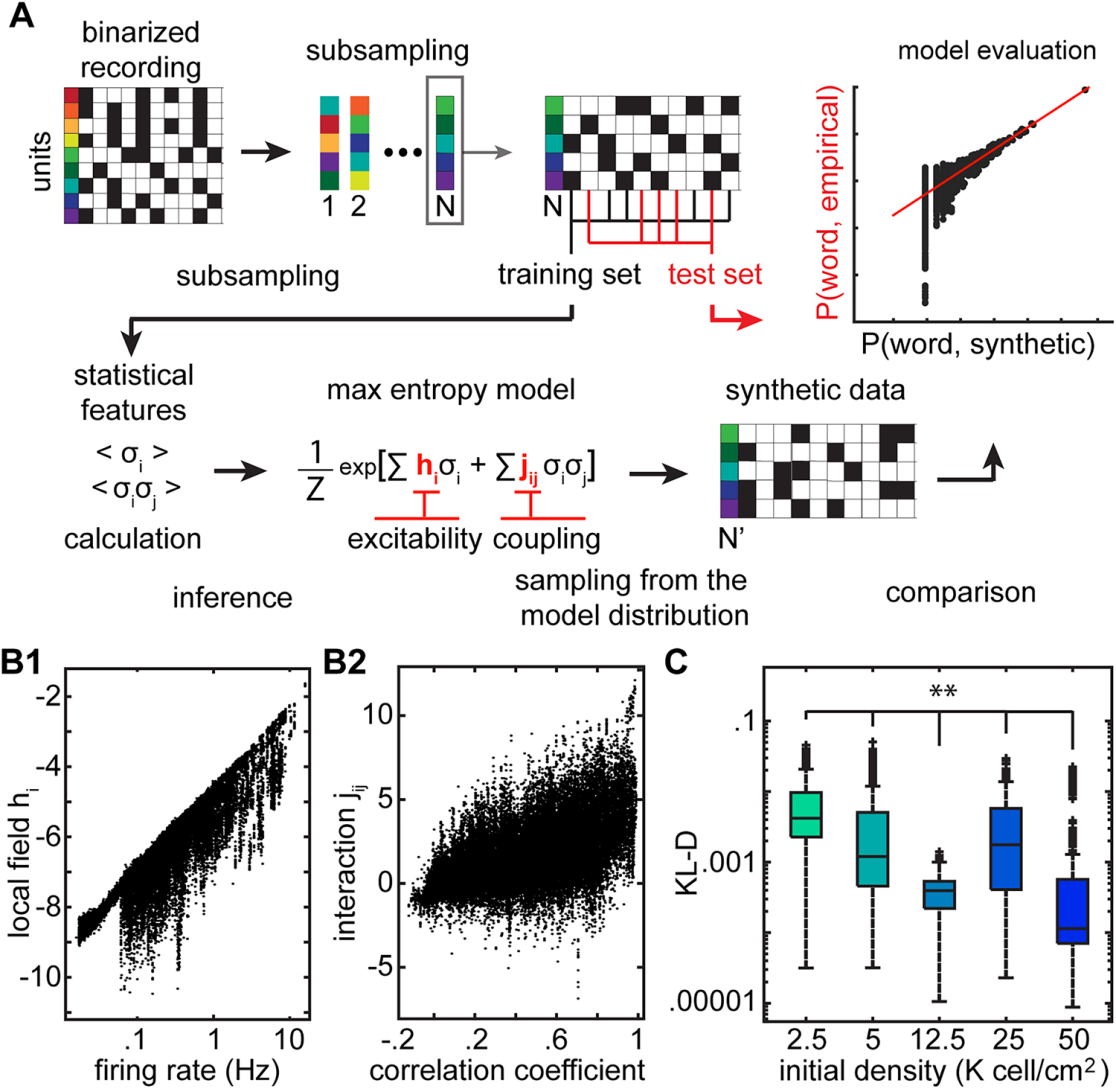
Maximum entropy models link the firing rates and pairwise interactions between the individual units to the global activity of the population*.* ***A***, Schematic representation of the maximum entropy model analysis from recorded data to model generation and fitting. First, the neural activity is binarized. Following this, we randomly selected subgroups of neurons from the total population of neurons that were recorded. The statistical features of the population activity (firing rate) and the pairwise interactions were extracted for each random subgroup. Only half of the time points were selected for training the model. A maximum entropy model was used to create synthetic data about the distribution of global patterns of activity across the population. This synthetic data was then compared with the empirical data to determine the goodness of fit of the model. ***B***, Pairwise model factors *h*_i_ (*n* = 24,258) and *j*_ij_ (*n* = 60,645) compared with firing rate of the corresponding units and correlation coefficient between the corresponding unit pairs. ***C***, Probability distribution of observing different network states estimated by the pairwise model. Performance of these estimations was evaluated by KL-D. Cultures coming from different initial density categories have significantly different KL-D distributions (initial densities 2.5, 5, 12.5, 25, 50 K cells/cm^2^ vitro, *p* < 0.0033). Statistics: two-sided Brunner–Munzel test with Bonferroni’s correction.

Complexity at the level of population activity implies complexity at the level of network organization. Networks that are structured in more complex ways would generate more complex patterns. While methods such as synaptic tagging and EM reconstructions could provide one way of linking structural complexity to complexity in the patterns of activity, alternative methods that offer benefits of scale have been developed to study this relationship. One approach to do this is to use a maximum entropy or pairwise Ising model from statistical physics (Schneidman et al., 2006; Tkačik et al., 2010). In the pairwise Ising model, one uses features describing elements of the circuit, such as the firing rate and the pairwise interactions to predict the distribution of the firing patterns at the population level. The intuition being that predictive power of the activity and pairwise interactions reflect the complexity of the network organization. These predictive models have been used to understand how the complexity of activity observed corresponds to the complexity of the underlying circuit in the retina (Shlens et al., 2006), olfactory bulb (Chockanathan et al., 2021), and the hippocampus (Meshulam et al., 2017; Chockanathan and Padmanabhan, 2021) and to uncover how changes in hippocampal circuits in a mouse model of amyloid pathology disrupt population activity (Chockanathan et al., 2020). In the pairwise model, the goal is to predict the empirical data (the distribution of activity patterns that arise at the level of the population) from the behavior of the elements (the firing rate of the neurons and the pairwise correlations of their activity) with as few assumptions as to what that structure is. As neuronal firing properties were stable over the duration of the recording, the Ising model allowed us to study the relationship between the microscopic feature of the network and the macroscopic distribution of activity under equilibrium conditions. To do this, we first subsampled different units in the detected population, for instance, five random units from a population of 9 neurons (Fig. 8*A*, top left). The activity of this population (spiking patterns) was divided into two parts, one used to determine the parameters of the pairwise model from which a distribution of activity patterns was generated (Fig. 8*A*, bottom) and the other used to assess the goodness of these predictions (Fig. 8*A*, top middle). The predictive model learned the features of spiking and correlation and optimized the parameters *h*_i_ (neural excitability parameter) and *j*_ij_ (neural interaction parameter), respectively, to determine what the distribution of activity patterns would look like (Fig. 8*A*, bottom). An example of a comparison of the empirical and model pattern probabilities is shown in Figure 8*A* (top right). The farther the points are from the unity line, the worse the prediction of the model.

First, we found a positive correlation between the firing rate and local field term *h*_i_ and between correlation and interaction term *j*_ij_ (Fig. 8*B1*,*B2*), as has been shown before (Schneidman et al., 2006). We then evaluated the extent to which the distributions of word patterns predicted by the pairwise Ising model matched the distributions of word patterns observed in the data by calculating the KL-D (Fig. 8*A*, top right). The higher the KL-D, the poorer the model is at predicting the data. We found that the pairwise model became significantly better at predicting the structure of the overall activity as the density increased (Fig. 8*C*). Our result suggests that lower density networks are more structurally complex than higher-density networks as the pairwise model was poorer at predicting the activity patterns in these low-density networks as compared with the higher-density networks.

## Discussion

Human monolayer cultures are a powerful tool for modeling human neuronal networks. Recent work has identified ways in which human iPSC-derived neuronal populations differ from cultured networks generated from primary cells of model organisms or human embryonic stem cells (Marchetto et al., 2013, 2019; Eyal et al., 2016; Sharf et al., 2022; van der Molen et al., 2026), suggesting that networks in iPSC-derived cultures may be unique in the activity patterns they generate. In studies using human iPSC-derived neuronal populations, cells are often cultured at different densities to model development and disease, including assessing the activity patterns generated using MEAs (Aghajanian, 2009; Marchetto et al., 2011; Brennand et al., 2012; Beltrão-Braga and Muotri, 2017; Vadodaria et al., 2020). Interestingly, it has been unclear how much of the differences in activity patterns seen across these experiments were due to the experimental protocols of differentiation, the scientific question and disease being modeled, or simply the density of the cultures. By varying one of the parameters of the culture, the density of neurons, we explored the impact of density on population activity. We found that several aspects of self-organization and functional coupling were changed and thus altered the patterns of activity that arose from the network. Our work also provides insight into dynamics in human networks where increasing evidence shows that these circuits are different from those of networks cultured from model organisms such as mice and rats (Hyvärinen et al., 2019; Estévez-Priego et al., 2022).

Although we cannot measure synaptic strength using extracellular MEAs, our data showing that the complexity of firing patterns reduces as density is increased suggest that these changes are due to the functional organization of the circuit, including the entropy or diversity of patterns of neuronal activity at the population level. Different firing rates and correlations can have significant impacts on the coding capacity. To solve if the reduction in entropy with increased cell density was a trivial consequence of either the correlation or the firing rate, we turned to pairwise maximum entropy models. Were the global differences in patterns of activity across cell culture of different densities only due to the correlations and firing rate differences, we would see no differences in the predictive power of the pairwise models for population activity. Instead, we found that the KL-D, which measures the error of our prediction, decreased as the cell density increased. This suggests that the complexity of iPSC-derived neuronal cultures reduce as the density increases. The simplest version of this reduction in complexity could be a network in which all the cells are connected to one another, which would reduce the entropy and reduce the KL-D (the error of the prediction).

The generalizability of this relationship must be tempered by several features specific to our experiments. First, our networks self-organize, and it is unclear if these relationships between density, firing rate, and entropy would be preserved if the networks assembled during development within the three-dimensional structure of the nascent nervous system where signaling cues that guide the formation of the nervous system are critical for synaptic connectivity (Lui et al., 2011). Additionally, much of development is influenced and instructed by patterns of activity that arise from external inputs, most often via sensory experience (Katz and Shatz, 1996), which we do not provide to our cultures.

Furthermore, another limitation of this study is that the coding capacity benefits of networks must be considered within the context that no specific computation is being optimized or performed by these networks. While the hypersynchronous activity that emerges as density is increased may be more reflective of circuits where a single dynamic behavior, for example, rhythmic firing, is required for a function, these networks perform no function and therefore the dynamics may be an emergent property of the connectivity. Future studies will be needed to identify both the mechanisms governing this emergence and the potential computational benefits of that activity. In contrast, a circuit with a comparatively low density of cells with connectivity that spans multiple spatial scales may more faithfully recapitulate the organization of a circuit in the associative region of the brain, where an increased coding capacity is essential for integrating diverse sensory and motor information to flexibly execute behaviors.

Our results here leave open critical questions about the mechanisms that may underlie the differences in activity associated with cell density that are areas of research for future investigations. We were only able to establish that the ratio of neurons to glia remained constant across cultures, but density may affect cell type heterogeneity, possibly in terms of the ratio of excitatory or inhibitory interneurons, or the subtypes of neurons that are present in different cultures (Tremblay et al., 2016; Deshpande et al., 2017; Bhaduri et al., 2020). Different in vitro differentiation protocols that produce different densities of cell types have been shown to result in different dynamics (Kang et al., 2017; Sarkar et al., 2018) and in some cases of neurological disorders where the density or type of cell being produced can influence the emergent dynamics of the cultures (Brennand et al., 2015; Deshpande et al., 2017; Amatya et al., 2019). Our study does not address if different culture densities can result in different distributions of cell types, which will be a future direction of interest. To this end, it will be important to understand how density and cell type are related when studying mechanisms of disease processes in future studies.

Additionally, we do not examine the spatial clustering in our cultures, which may also affect neuronal activity. In studies where spatial clustering has been patterned in vitro using cells from animal models including embryonic cells, there is clear evidence of differences in network activity and dynamics (Yamamoto et al., 2018; Hörberg et al., 2022). Previous studies have shown differences in cell proliferation and migration across different species that may also affect the extent to which human neurons may cluster in vitro (Marchetto et al., 2019) as compared with other species which will be an important area for future study. As a result, we cannot disambiguate whether distinct densities cause differences in clustering. Additionally, differences in clustering can shape activity patterns as has been shown in computational models (Litwin-Kumar and Doiron, 2012).

As future experiments may focus on a mechanistic dissection of what impacts network activity, this manuscript provides metrics for studying these features including how the firing rate and the pairwise correlations between active neurons shapes the global structure and patterns of activity. These approaches may thus provide metrics for future experimental studies investigating mechanisms in human iPSC-based models of disease. In this regard, we present here not only a result about the relationship between the density of neurons in human cultures and the structure of activity that can be generated, but a way of investigating the global structure of activity more broadly in multiple applications related to human neural cultures ranging from development to the neurobiology of brain disorders.

## Synthesis

Reviewing Editor: Arvind Kumar, KTH Royal Institute of Technology Decisions are customarily a result of the Reviewing Editor and the peer reviewers coming together and discussing their recommendations until a consensus is reached. When revisions are invited, a fact-based synthesis statement explaining their decision and outlining what is needed to prepare a revision will be listed below. The following reviewer(s) agreed to reveal their identity: Ulrich Egert.

Synthesis

First of all my apologies for the slow processing of this manuscript. After reading the reviewers' reports and having short discussion on their comments we reach the conclusion that some of the key concerns of the first reviewer still have not been resolved.

The main point of contention is whether 'density' is "the framework" that summarizes the main differences across networks. There are other confounding factors that have not been studied e.g. substrate adhesion, clustering. Clearly density depends on such parameters. So it seems that the main claim of the manuscript is not really justified given the data and analyses. As one the reviewer pointed out "Overall the discussion is not at all balanced and overinterprets the data, with just a slight moderation." And I agree with this.

Therefore, I would like to ask you to revise the manuscript. Further comments from the reviewers are appended below.

Reviewer comments:

In the resubmitted manuscript the authors adequately address several of the concerns raised by the reviewers of the previous version. Some critical aspects, however, are not resolved and additional significant issues become apparent. One fundamental problem mentioned before is the causal link between density and activity dynamics of the networks. The authors now address this along two main lines: immunohistochemical (IHC) staining for cell types (Fig. 1) and an indirect assessment of structural similarity based on estimates of neuron positions (Fig 3).

Neither of the lines of analysis provides sufficient support for the claim that cell density is the critical and distinguishing factor for the activity in the networks. Several critical aspects shaping network activity reported in other publications, in particular visible differences in network structure, are not addressed. Density could well be just one aspect influencing those indirectly.

Major critique

Composition of the networks. IHC stains are used to identify neurons (MAP2, mainly marking somata and dendrites) and astrocytes to address a critique by other reviewers. The stain for GABA mentioned in the caption for Fig. 1 is neither visible in Fig. 1A nor indicated in its legend. So potential heterogeneity of cell types is not resolved and might differ across network densities. In any case, MAP2-IHC does not distinguish well between different subtypes of neurons and thus the question about density-dependent heterogeneity remains unanswered.

Furthermore, estimating visually from the whisker-plots in Fig. 1B, the ratio of means of cells at 8 weeks vs. cells seeded decreases considerably with increasing seeding density (~18.0, 19.0, 9.2, 6.8, 3.9), indicating that there might be additional factors contributing to differences in spike activity besides density.

At third aspect is the network morphology apparent in 1A. Networks with initially 2.5, 5, and 25 k cells/sqcm are clearly clustered, for 12.5k and 50k this cannot be determined well from the images provided. Clustering, however, has a prominent impact on activity, as shown for example in several papers from the labs of J. Soriano, U. Egert or S. Marom. Intra-cluster connectivity is higher than inter-cluster connectivity, which is particularly pronounced in low density networks. This difference decreases with increasing overall network density. So dense networks with more homogeneous connectivity have very different activity dynamics compared to sparse networks with heterogeneous connectivity. However, this is due to their structure and at best only indirectly to (local) density (see, for example, Okujeni et al. 2017). There is further relevant work from the same lab. For the impact of clustering created by patterned substrates see work from J. Soriano's lab. Even quite coarse subpartioning will affect activity propagation (see Marom lab). Aspects of this already indicate that pattern variability would decrease in larger and putatively more homogeneous networks because of more comprehensive recruitment and synchronization. These publications point to a more mechanistic explanation for the observations in this ms than just neuron density. While all of them have been done on primary cultures, there is no indication that this should be fundamentally different in hIPSC derived networks.

To further investigate structural differences, the authors localize neuron positions by triangulation based on spike amplitudes after spike sorting. As I understand this process, the results are then meant to support the claim that the network structure does not change with network density. There are two issues with this. Firstly, while amplitude does scale with distance, for the triangulation the spike shape must not change. Changing shapes across two electrodes indicate a different distribution of the current sinks and sources and introduces ambiguity of spike size, as will different dendritic morphologies (see e.g. Lindén et al. 2010, Fig. 1 and Lindén et al. Neuron 2011 Fig 3, Jäckel et al JNphys 2012 Fig 4). According to Jäckel et al., extrac. spikes are largest at soma or axon hillock but elsewhere contributions mixed in from dendritic source can diminish spike amplitudes and distort direction-dependent decay functions. More positive dendritic spikes might even be larger further away from the soma than closer to it. To illustrate this, sampling spike amplitudes from the soma towards the apical dendrite of a pyramidal cell or a Purkinje cell will produce a different decay function than going perpendicular to this axis.

Unfortunately, the methods section on MEA recordings is very minimalistic, does not provide any details about the MEAs beyond "12-well MEA plates" from Axion and is definitely insufficient. Standard information needed to assess the electrophysiology or reproduce it is missing: what were impedance, electrode pitch, electrode diameter, bandpass filter during recording? Since the sampling rate was a quite low at 12.5 kHz, the Nyquist frequency would be 6.25 kHz with a suitable low-pass. Because of the filter's roll-off, it would likely have a cut-off frequency much lower than that. Hence, using a bandpass filter during recording and a second stage in post-processing creates a considerable phase shift and hence a distortion of the spike shapes. The high-pass cut-off used during recording is not indicated but tends to have a more pronounced effect on spike shapes. Generally speaking, the more one filters, the more similar the spikes become and the more difficult their separation during sorting. So, given the lack of information, it may be that more units were present in the recording at an electrode than were isolated.

Spikes sorting issues have been discussed already with the previous version of the ms and most have been addressed in this submission. There is, however, another aspect that comes into play. As is visible in Fig 4A, spikes are often concentrated in bursts. At least in my experience with spike sorting, separating units becomes increasingly problematic as bursts become more compact, i.e. when synchronization of neurons around an electrode increases, because of the increasing probability for overlapping spikes. This can result in a bias to discard such spikes that cannot be clearly assigned to one unit (or assign them to a separate unit), which seemingly decreases the firing rate. Did the fraction of spikes in bursts change with density? Please explain how you ensured that the fraction of overlapping and excluded spikes did NOT increase with the degree of synchronization and how you handled such compound spikes in the further analyses. Synchronized spontaneous bursting events in dense, more homogeneous networks are often more compact than in sparse and/or clustered networks. Such a bias would therefore work in the direction of decreasing firing rates as in Fig. 5. In contrast, it might reduce the decrease of word entropy with increasing density.

The subsampling used in the entropy analysis to compensate for different yields of electrodes detecting spikes assumes that the probability to find spikes at some position scales directly with density. This would only be true if neurons are evenly distributed and dendrites are similar. Judging from Fig 1A, the first assumption is not correct. Clustering will lead to a bias in the subsampling and this bias will depend on density since this influences clustering. While in clustered networks electrodes with spikes will necessarily be below clusters, they might sit anywhere in homogeneous networks. This may not show up in entropy measures and underestimate the contribution of clustering to activity patterns.

Minor

L417: The debate on whether homeostatic scaling depends on firing (output) or synaptic input has been going on for quite some time. Most publications cannot distinguish this. Other detailed analyses, e.g. Fong et al Nat Com 2015, Barral &Reyes Nat Neurosci 2016, Okujeni &Egert eLife 2019, provide support for input-dependent homeostatic processes. Changing firing rates would only be consequence of this. The statement starting L417 should thus be moderated and include this perspective.

Fig 1A caption: Please remove the reference to GABA staining or provide micrographs that include it.

Fig 2E: The coloring suggests that the PCA in E1 and the waveforms in E2 correspond to the units in 2C. The PCA clusters in 2C looks different though than the one in the center of E1, whereas the spike shapes in E2 look like those in 2C. Please show the matching distributions and spike waveforms across panels or explain the differences in the caption.
